# PacBio Amplicon Sequencing Method To Measure Pilin Antigenic Variation Frequencies of Neisseria gonorrhoeae

**DOI:** 10.1128/mSphere.00562-19

**Published:** 2019-10-02

**Authors:** Egon A. Ozer, Lauren L. Prister, Shaohui Yin, Billy H. Ward, Stanimir Ivanov, H Steven Seifert

**Affiliations:** a Department of Microbiology-Immunology, Northwestern University Feinberg School of Medicine, Chicago, Illinois, USA; b Department of Medicine, Division of Infectious Disease, Northwestern University Feinberg School of Medicine, Chicago, Illinois, USA; c Department of Microbiology and Immunology, Louisiana State University Health Sciences Center-Shreveport, Shreveport, Louisiana, USA; University of Michigan—Ann Arbor

**Keywords:** antigenic variation, Pacific Biosciences sequencing, amplicon sequencing, gene diversification

## Abstract

Gene diversification is a common mechanism pathogens use to alter surface structures to aid in immune avoidance. Neisseria gonorrhoeae uses a gene conversion-based diversification system to alter the primary sequence of the gene encoding the major subunit of the pilus, *pilE*. Antigenic variation occurs when one of the nonexpressed 19 silent copies donates part of its DNA sequence to *pilE*. We have developed a method using Pacific Biosciences (PacBio) amplicon sequencing and custom software to determine pilin antigenic variation frequencies. The program analyzes 37 variable regions across the strain FA1090 1-81-S2 *pilE* gene and can be modified to determine sequence variation from other starting *pilE* sequences or other diversity generation systems. Using this method, we measured pilin antigenic variation frequencies for various derivatives of strain FA1090 and showed we can also analyze pilin antigenic variation frequencies during macrophage infection.

**IMPORTANCE** Diversity generation systems are used by many unicellular organism to provide subpopulations of cell with different properties that are available when needed. We have developed a method using the PacBio DNA sequencing technology and a custom computer program to analyze the pilin antigenic variation system of the organism that is the sole cause of the sexually transmitted infection, gonorrhea.

## Abstract (precis)

Diversity generation systems are used by many unicellular organism to provide subpopulations of cell with different properties that are available when needed. We have developed a method using the PacBio DNA sequencing technology and a custom computer program to analyze the pilin antigenic variation system of the organism that is the sole cause of the sexually transmitted infection, gonorrhea.

## INTRODUCTION

Antigenic variation (Av) describes high-frequency, reversible gene diversification processes resulting in the expression of many different forms of a gene product. Av is a common process in many microbial pathogens, including viruses and bacteria, and eukaryotic parasites (1–4). Gene diversification allows for stochastic population heterogeneity, which can be beneficial at the population level when selection occurs (5). While the name suggest that these systems are in response to immune surveillance, the variation can be useful for both immune and other function selection on the population level.

Neisseria gonorrhoeae is a human-specific organism and the sole causative agent of gonorrhea. During infection, a robust innate immune response comprised of recruited polymorphonuclear cells (PMNs) and macrophages localize to the site of infection (6, 7). PMNs are the most common immune cell recruited during infection, and much of the interactions with PMNs such as recruitment and signaling have been established (8). In addition to PMNs, macrophages have been isolated from acute infection sites and N. gonorrhoeae have been shown to modulate apoptosis and stimulate the release of cytokines and antimicrobial peptides (9–11). N. gonorrhoeae can survive in the presence of macrophages; however, much remains unknown about how N. gonorrhoeae interacts with macrophages.

**FIG 1:**
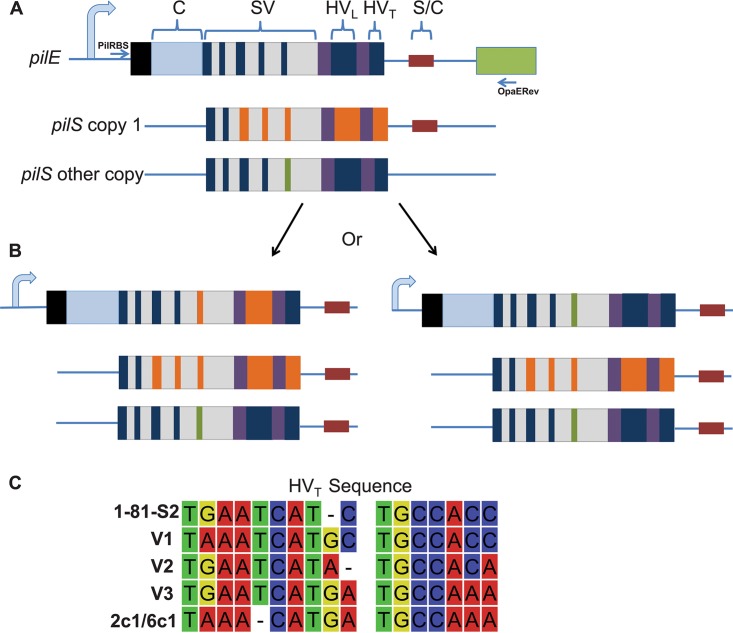
Diagram of *pilE* gene and Av process. (A) Cartoon of the *pilE* gene and downstream *opa* gene (green). The 7-amino-acid signal sequence necessary for proper localization of the protein (black box) is at the N terminus of the preprotein. The constant region (“C” [light blue]) is not present in in any silent copy. The semivariable (SV) region has short variable regions with single-amino-acid substitutions (navy) in between conserved sequences (gray). The hypervariable loop (HV_L_) is located between the conserved *cys1* and *cys2* regions (purple), and the gene ends with the hypervariable tail (HV_T_). The 3′ untranscribed nor translated SmaCla region (S/C) (red) is found in both *pilE* and the 3′ end of all *pilS* copy 1 donors but not *pilS* copies 2 to 6. (Only one locus has six copies.) The primers used for amplification, PilRBS, and OpaERev are shown in blue and are not present in any silent copy. (B) Gene conversion from a *pilS* copy to *pilE* can occur anywhere within the variable regions bordered by regions of microhomology. In the left example, a portion of the SV and the entire HV_L_ sequence of the top *pilS* copy 1 donor replaces the similar *pilE* sequences, but the *pliS* copy remains unchanged. In the right cartoon, the bottom *pilS* copy donates a small region of variant SV sequence. (C) Av events occurring at or near the HV_T_ can result in a mosaic sequence of the tail. This can occur when one DNA strand is parental and the other is a DNA strand is from a silent copy, such as 2c1, creating a heteroduplex of DNA. The mosaic sequences seen in this assay were similar to the parental (1-81-S2) and the 2c1/6c1 silent copies. The same three HV_T_ mosaic sequences (V1, V2, and V3) were most common in both *recA6* +IPTG samples and both FA1090 pools. Sequences were displayed using the CLC Sequence viewer (Qiagen).

To avoid adaptive immune recognition, one of the surface-exposed variable proteins, the type IV pilus, varies through conversion of the gene encoding the major pilin subunit, PilE (12, 13). The type IV pilus is required for establishing infection, as all human isolates of N. gonorrhoeae are piliated, but the role of nonpiliated bacteria during infection is unknown (14–17). Therefore, it is important that this essential factor changes throughout infection to avoid immune detection. During pilin Av, a portion of one or more donor silent copy sequences replaces part of the *pilE* gene in a nonreciprocal, homologous recombination process (18, 19). There are 19 N. gonorrhoeae silent *pilS* copies found at various loci throughout the strain FA1090 genome (20). Any portion of the recombining silent copy, from the entire variable region to a single base can be transferred into the *pilE* locus. Recombination only requires regions of microhomology at the ends of the new sequence, and after recombination, the donor silent copy sequence remains unchanged (Fig. 1). Pilin Av requires many conserved common recombination and repair factors that process gap repair and double-strand breaks (21). Inactivation of some required factors, such as RecA (22), RecO, RecR, and RecG, completely abrogate pilin Av (23–25), while mutation of other factors, such as RecQ, Rep, and RecJ reduce pilin Av frequencies (24, 26–29).

Additionally, there are two *cis*-acting factors that function in pilin Av. An alternate DNA structure called a guanine quadruplex (G4) forms upstream of the *pilE* gene (30, 31). Mutation of any of the individual GC base pairs necessary to form the *pilE* G4 structure prevents pilin Av, while mutation of any of the TA base pairs within the sequence, which are not required to form the structure, allows normal levels of pilin Av (31). Pilin Av also depends on a promoter located adjacent to the G4-forming sequence that initiates transcription within the G4 sequence resulting in a small, noncoding RNA that can only function in *cis* (32, 33).

The ∼500-bp *pilE* gene contains a promoter, ribosome binding site, and conserved 5′ coding region (constant region), which is not found in any of the silent copies (Fig. 1). From bp 150 to 360, there is a semivariable (SV) region, which contains short regions of homology interspersed with short regions of variation that are also templated in one or more silent copies. *cys1* and *cys2* are conserved DNA sequences, which encode the disulfide bridge forming cysteines within the PilE protein (34). Located between the conserved cysteine regions is the hypervariable loop (HV_L)_, which shows the largest amount of protein and DNA sequence diversity (Fig. 1). Finally, the *pilE* hypervariable tail (HV_T_), from bp 477 to 492, is also highly variable in length and sequence (18). After the coding sequence, the conserved 65-bp Sma/Cla repeat is also found in *pilE* and at the end of all silent loci and provides downstream homology for recombination (35–37).

Pilin Av leads to a change in the *pilE* sequence with anywhere from the entire variable sequence replaced to as little as 1 bp altered. We are unable to determine whether surrounding regions of homology are also transferred during recombination events because the starting and ending sequence would be identical. Pilin Av has been analyzed using a variety of methods, including Southern Blot hybridization for the loss or gain of variable sequences, quantitative reverse transcription of specific variable sequences, enumerating the production of nonpiliated progeny, and determining the pilus-dependent colony morphology changes (PDCMC) over time (23, 35, 38–42). All of these methods have limitations in reproducibility and/or are affected by small changes in the growth rate. A Sanger sequencing method was developed that allowed quantification of pilin Av frequencies independent of growth rate but was limited by the low numbers of events that could be reasonably interrogated (38). The Sanger method used a N. gonorrhoeae strain encoding an inducible *recA* allele to regulate pilin Av and determined the pilin Av rate of 6.3 × 10^−3^ per CFU per generation for strain FA1090 *recA6 pilE* allele 1-81-S2 (38). A Roche 454 long-read sequencing method was developed to measure pilin Av frequencies that allowed large populations to be screened that also was not affected by growth rate (28). High-throughput sequencing with long read lengths allows for the continuous sequencing of the entire variable region in one read. In contrast, short-read technologies such as Illumina would allow determination of changes in the population, but would not be sufficient for identification of individual variants (43). Roche 454 sequencing technology is no longer available, so there is a need for new methods of pilin Av analysis with other long-read sequencing technologies.

PacBio single-molecule, real-time (SMRT) sequencing has been used to determine VslE Av frequencies in Borrelia burgdorferi (44). VlsE Av is required for persistent colonization of the host because it creates a heterogeneous population that cannot be cleared by the host immune system (45). PacBio amplicon sequencing entails ligating a single-stranded hairpin adaptor to each end of the amplicon, which, when denatured, creates a circular single-stranded DNA (ssDNA) molecule to be sequenced. However, PacBio sequencing is not very accurate, with error rates around 15% per base and with base mismatch and insertion/deletion errors being common (46). With circular consensus sequencing (CCS) on PacBio, each amplicon circle can be sequenced continuously to generate reads from the same template sequence several times over, leading to improvement on the accuracy of base calling. PacBio CCS was successfully used to characterize and measure B. burgdorferi VslE variation (47).

We developed a method to determine *Neisseria* pilin Av frequency using PacBio CCS and used this method to analyze pilin Av in strains of N. gonorrhoeae that have previously been tested by other methods of analysis and the FA1090 common lab strain, which has never been systematically analyzed before. To aid in this analysis, we developed bioinformatic methods and software to annotate variants in reads and account for the residual errors in PacBio sequencing. We report FA1090 Av frequencies in several conditions and compare our new method to previously developed methods of analysis. Finally, we show the utility of this method by measuring pilin Av frequencies of N. gonorrhoeae associated with human macrophages.

## RESULTS AND DISCUSSION

### Optimization of conditions for PacBio sequencing.

**FIG 2:**
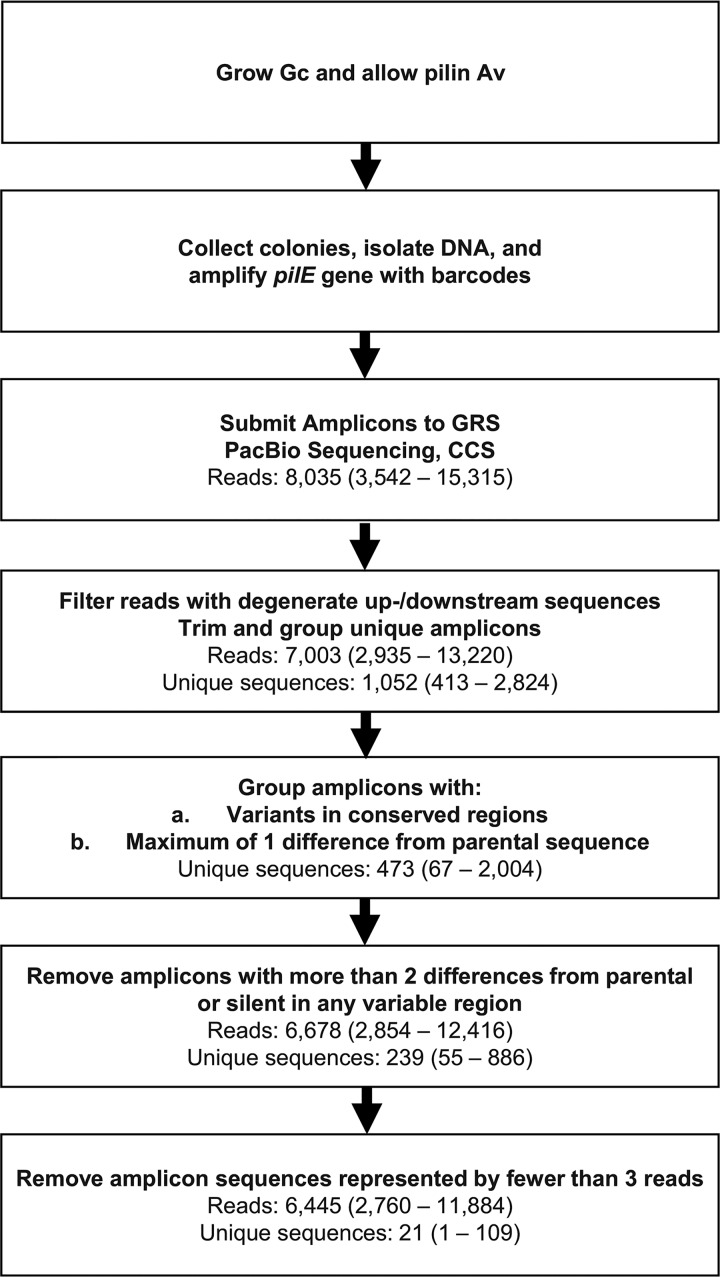
Flow chart of protocol and results from amplicon read processing. Strains were grown for 22 h, and colonies were pooled to isolate genomic DNA. The *pilE* gene was amplified with primers containing a specific barcode for each condition or sample. The PCR was gel purified and sent to the Genomics Resource Center for library preparation. The reads were then demultiplexed and analyzed using SwitchAmp as described in Materials and Methods. The flow chart describes the filtering steps used for amplicon read processing in SwitchAmp. The average number of reads in each step is displayed below each step, with the range in parentheses for each pool.

To enable measuring of pilin Av frequencies by PacBio sequencing, strains were grown for 22 h on GCB plates, which results in ∼19 to 20 generations (28). The isopropyl-β-d-thiogalactopyranoside (IPTG)-inducible *recA6* strains were grown in duplicate on solid medium with IPTG to allow for pilin Av. All of the strains tested had similar growth rates, so all N. gonorrhoeae strains were harvested at the same time point (28). Between 1,041 and 1,255 colonies were pooled, and genomic DNA was extracted (Fig. 2).

False “variants” can be produced by *in vitro* recombination during PCR amplification when an extension intermediate primes a different silent copy extension intermediate, producing a hybrid sequence of parent and silent copy (28, 48). This has also been seen in other systems, such as analysis of drug-resistant allele variants of HIV (49). To limit these PCR artifacts, low genomic template (1 ng), a high-processivity polymerase (Phusion Hot Start Flex), and the touchdown PCR cycles were used. Touchdown PCR initiates with high annealing cycle temperatures, which are slowly lowered each annealing cycle to ensure specific primer binding of the correct region. Using all these techniques, PCR recombination was reduced to background levels (Table 1). PacBio barcodes (see Table S1 in the supplemental material) were used to differentiate the samples, and to minimize PCR cycles, the barcodes were added to the primers for the *pilE* gene, OpeERev and PilRBS (Fig. 1) (50, 51). Each pair of barcoded primers was tested, and any pairs showing aberrant products were not used. After PCR, the products were gel purified from gels without ethidium bromide by staining a reference lane to localize the product, and DNA was extracted with the QiaQuick gel extraction kit without heating the gel slices. The University of Maryland Genomic Resource Center conducted the further steps, including PCR purification using solid-phase reversible immobilization and quantification by Qubit. The pooled samples were prepared for sequencing with the SMRTbell library kit from PacBio and run on the Sequel system (Fig. 2). After sequencing, reads were demultiplexed, and consensus sequence was determined by SMRT Analysis software with a read confidence of 90, minimum read of three passes, and minimum read length of 50. The average amount of total sequence generated per condition was 6.32 Mbp (range 2.76 to 11.91 Mbp). The average read length was 786.7 bp, with a median read length of 789 bp. In all samples, including those from total macrophage/N. gonorrhoeae DNA there was sufficient number of reads per sample to have a measure of pilin Av frequencies. Read characteristics and NCBI accession numbers are shown in Table S2 in the supplemental material.

**TABLE 1:** Pilin Av frequencies of strain FA1090 *recA6* 1-81-S2^*a*^

Strain type	PacBio							*P* value vs − IPTG	454 method				
	Pool A		Pool B		*P* value for A vs B	Total			Strain	Pool A		Pool B	
	No. of reads	% Av	No. of reads	% Av		No. of reads	% Av			No. of reads	% Av	No. of reads	% Av
*recA6* − IPTG	8,881	0.17	ND	ND		8,881	0.17	NA	Av deficient	96,993	0	ND	ND
*recA6* + IPTG	4,001	4.65	11,884	6.70	3.9E−06	14,903	6.18	1.3E−117	*recA6* + IPTG	6,495	10.6	5,977	10.8

a Pilin Av frequencies from PacBio Sequencing and SwitchAmp analysis were compared to those reported by Rotman et al. (28) using the 454 method and a different computational program. The pilin Av frequency was calculated by dividing the number of *pilE* variant reads by total *pilE* reads after filtering for quality. *P* values were calculated by chi-square tests comparing Av read proportions between biological replicates (A versus B) or proportions of Av reads between summed totals of reads under each condition. The number of reads listed is the final number of reads after all filtering steps listed in Fig. 2. ND, not determined; NA, not applicable.

10.1128/mSphere.00562-19.1 TABLE S1 Primers used in this study. Listing of the barcoded primers used in this study for PacBio sequencing. Download Table S1, PDF file, 0.1 MB. Copyright © 2019 Ozer et al. 2019 Ozer et al. This content is distributed under the terms of the Creative Commons Attribution 4.0 International license.

10.1128/mSphere.00562-19.2 TABLE S2 Read statistics. The strain names, accession numbers, reads, and number of sequences used during each step of filtering and analysis. Download Table S2, PDF file, 0.1 MB. Copyright © 2019 Ozer et al. 2019 Ozer et al. This content is distributed under the terms of the Creative Commons Attribution 4.0 International license.

### SwitchAmp program for variant analysis.

To analyze the sequencing reads, we identified all regions that can differ between the 1-81-S2 *pilE* sequence and all the possible changes that could occur from any of the 19 silent copies (see Table S3 in the supplemental material), since all variation events should be templated from a silent copy. For all samples, the average number of CCS reads generated per amplicon set was 8,035 reads (range, 3,542 to 15,315) (Fig. 2 and Table S2). On average, 13% of reads (range, 9.8 to 20.4%) were filtered for exceeding a maximum of two mismatches in either the upstream and/or the downstream sequences flanking the *pilE* gene sequence. This analysis resulted in an average of 7,003 amplicon sequences in total per sample (range, 2,935 to 13,220). Amplicon sets were filtered to group all amplicons with either isolated conserved region mismatches or fewer than 2 base mismatches across variable regions with the parental sequence. Amplicon sets were then further filtered to remove sequences that had more than 2 base differences from parental sequence or any silent copy. This filtering resulted in an average of 6,678 sequences per read set (range, 2,854 to 12,416). We chose to remove amplicon sequences represented by fewer than three reads in each set to reduce potential error from low-frequency variants resulting from sequencing errors. Using this cutoff resulted in removal of an average of 93.8% of unique sequences (range, 87.2 to 98.9%) in each read set; however, those unique sequences only represented 3.5% of all previously filtered reads (range, 1.0 to 13.2%). These results indicate that the SwitchAmp algorithm effectively identifies and characterizes high-quality Av sequences from long-read amplicon sequencing experiments. On average, we excluded 19.8% of reads from our analysis through all of these filtering steps. Although the error rate of PacBio sequencing is higher than other methods of deep sequencing, we believe that through our successive filtering steps we have removed the sequences that do not represent true biological sequence variants.

10.1128/mSphere.00562-19.3 TABLE S3 Alignment of variable *pilE* and *pilS* sequences. The top row shows the nucleotide position within *pilE* of each variable region. There are 37 regions of variation separated by short regions of microhomology. The sequence present in each silent copy is located below each *pilE* position. The parental 1-81-S2 sequence is shown at the bottom. This table was used as the template for the SwitchAmp program and would have to be modified for a different starting *pilE* variant or a different diversity generation system. Download Table S3, PDF file, 0.09 MB. Copyright © 2019 Ozer et al. 2019 Ozer et al. This content is distributed under the terms of the Creative Commons Attribution 4.0 International license.

### Pilin Av frequencies in IPTG-regulated *recA6* strains.

Strains with the IPTG-regulatable *recA6* allele were grown for 22 h as described previously (28). Without RecA induction, we measured 0.17% pilin Av (Table 1). We have repeatedly analyzed pilin Av in this strain without IPTG induction and have never recorded a true pilin Av event over many studies (22, 29, 52). Therefore, we conclude that these variant reads are the result of sequencing errors being recorded as true events or alternatively the result of PCR recombination. The pilin Av frequencies with IPTG induction of RecA were 4.65 and 6.70% in two biological replicates (pools A and B). The 2% difference in the frequency of pilin Av measured between the two biological replicates is most likely due to the stochastic nature of pilin Av and the fact that events that occur early will be overrepresented in the population. We employed chi-square statistical analysis to this data set and found that there is a significant difference between the two biological replicates. These data highlight the importance of biological replicates and demonstrate that PacBio can be used to measure pilin Av in N. gonorrhoeae. These pilin Av frequencies are lower than reported using 454 sequencing and a different analysis method (Table 1). The 454 sequencing had a different error rate (0.49% per base) (53) and in the absence of IPTG had a higher background rate of about 1%. With different error rates, we may be discounting actual variants if there are errors in other regions of the *pilE* gene and the sequence is then discarded. The higher error rate of PacBio sequencing is a drawback of this technology and requires stringent filtering programs. The two methods also used different computational analysis methods, which call variants differently.

### Pilin Av frequencies in an unregulated FA1090 strain.

All previous pilin Av sequencing studies have used the *recA6* strain to start with a uniform population and to limit Av to a specific number of generations. Measuring pilin Av frequencies of FA1090 with an unregulated *recA* gene has never been reported. The difficulty with measuring Av in FA1090 without the *recA6* allele is that *pilE* is constantly varying during growth and the experiment cannot start with a single variant. Therefore, it is likely that early Av events will occur and predominate in the population. We used this PacBio method to measure Av in FA1090. N. gonorrhoeae strains were grown in duplicate overnight on solid medium from freezer stocks for 18 h. Several single progenitor colonies were each plated onto solid medium and grown for 22 h. In parallel, the *pilE* of each progenitor colony was sequenced by Sanger sequencing and only progenitors with the *pilE* allele 1-81-S2 were processed for PacBio sequencing. Sanger sequencing only gives a population-level sequence of the most common base at each position and since Av frequencies are approximately 10% across the variable regions of a gene (35), this low-frequency variation at a population level cannot be detected by standard sequencing, and we are certain that there was always a population of variants that arose during the growth of the progenitor colony.

As anticipated, the continual pilin Av frequencies measured for FA1090 were higher than those of *recA6* strains (Table 2). The pilin Av frequencies of the two FA1090 biological replicates were 17.90 and 17.40% (pools A and B), and these two replicates were not significantly different. Mutation of the G4-forming sequence or the promoter of *pilE* G4 small RNA (sRNA) (*gar*), which are both required for Av (31, 32), produced pilin Av frequencies of 0.21 and 0.1%, respectively (Table 2).This level of variation is similar to the pilin Av frequency of the *recA6* strain without *recA* induction (Table 1). The lack of variants (less than 15 reads in each sample) in the *recA6*-IPTG strain, the G4 mutant strain, and the *gar* promoter mutant shows that our stringent filtering has excluded sequencing errors and almost all of the reported variants represent true variant sequences. Mutation of the −35 sequence of the G4 sRNA promoter (*garP*_−_*_35_*) in the same FA1090 background showed reduced levels of pilin Av of 6.13 and 5.73%, which is consistent with the reduced levels of *gar* RNA produced when the −35 sequences was mutated. (Table 2) (33).This reduction in pilin Av frequency is significantly reduced compared to FA1090 by chi-square analysis (Table 2). We assume that these levels of pilin Av represent steady-state levels under these growth conditions.

**TABLE 2:** Pilin Av frequencies of FA1090 strains^*a*^

Strain type	Pool A		Pool B		*P* value for A vs B	Total		*P* value vs FA1090
	No. of reads	% Av	No. of reads	% Av		No. of reads	% Av	
FA1090	7,296	17.61	5,396	16.9	0.27	12,692	17.3	NA
G4 mutant	7,252	0.21	ND	ND	NA	7,252	0.21	2.4E−298
FA1090 *garP_−10_*	4,707	0.1	ND	ND	NA	4,707	0.13	3.1E−200
FA1090 *garP_−35_*	4,522	6.13	4,434	5.73	0.45	8,955	5.9	3.7E−135

a Av frequencies of the FA1090 strain were calculated similarly to Table 1 using PacBio sequencing and analysis with SwitchAmp. *P* values were calculated by chi-square tests comparing Av read proportions between biological replicates (A versus B) or proportions of Av reads between summed totals of reads relative to the parental strain (FA1090). ND, not determined; NA, not applicable.

These results demonstrate that PacBio amplicon sequencing can measure differential pilin Av frequencies in FA1090 strains without the use of the inducible *recA* construct. Both biological replicates of FA1090 were very similar in Av frequency, but the *recA6* strains did have some variability between replicates, highlighting the need for replicates. Since FA1090 populations can undergo Av at any time point during the experiment, there is potential for a “jackpot” event to occur very early in growth and result in a large portion of the bacteria containing the variant sequence. Therefore, it is necessary to perform biological replicates to verify that Av frequency measurements are consistent. This method may not be able to differentiate small differences, because there is still variation between biological replicates.

### Analysis of silent copy donors.

We determined which silent copies were used during pilin Av, and if any of the mutants analyzed had different patterns of donor silent copy usage. Previously, no mutation has been shown to alter silent copy choice (28, 38). The SwitchAmp software we developed identifies the most common silent copy sequence among all the regions of variation in each amplicon sequence. This analysis can allow for the identification of the donor silent copy in a variant, because if one region has a sequence change common among many silent copies, and the next region also has a change that matches a single silent copy, the most likely donor copy can be inferred. For example, if in one read, one variable region has a sequence that is identical in silent copies 1c1, 1c2,1c3, and 1c4 and the next downstream variable region has a sequence that is found only in silent copy 1c2, then the most likely donor was 1c2. SwitchAmp also provides a table of the silent copy choice for each variable region in each variant sequence, so a more in-depth analysis can be performed with manual inspection.

**FIG 3:**
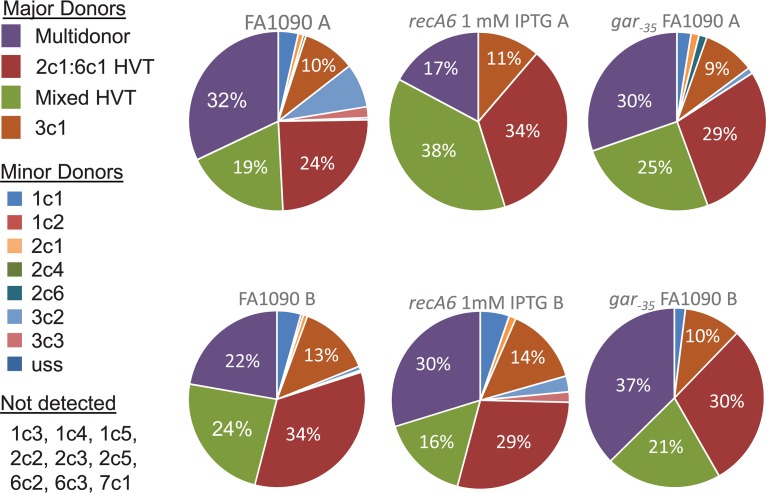
Donor silent copies. The most common silent copy found in each variant sequence was determined for strains FA1090, *recA6* plus 1 mM IPTG, and FA1090 *gar_−35_*. “Multidonor” indicates that the changed sequence was common to multiple silent copies, and there was no dominant silent copy. HV_T_ is the hypervariable tail, or var37 in our analysis. This is the most common region of variation. Mixed HV_T_ indicates that the tail contained a mosaic sequence. The minor silent copies were used at a much lower rate, and some silent copies were not used in our analysis. A and B refer to the biological duplicates of pools A and B for each condition.

There were four variants most commonly seen among our parental strains (Fig. 3). The most common variant in most strains was the change to the identical *pilS2* copy 1 (2c1) or *pilS6* copy 1 (6c1) in variable region var37, which is also called the hypervariable tail (HV_T_) (54). The next most frequent donor was *pilS3* copy 1 (3c1), mostly in region var32, which is also called the hypervariable loop (HV_L_) These donors are the same as the most frequent report previously using this same strain and starting *pilE* sequence (12, 18, 28, 38, 55). Another category of variants was multidonor, which refers to sequence changes common to multiple silent copies. There are many regions in the silent copies that have shared sequences; therefore, the exact donor sequence cannot be determined. Additionally, a double recombination event could also be part of this population when both recombination regions have a similar length: for example, if regions 3 and 4 have sequence donated from the 3c1 copy and regions 15 and 16 have sequence from the 1c1 copy, the output would include both sequences, and be termed “multidonor” for multiple potential donors. However, for example in a different variant, if regions 3, 4, and 5 have sequence donated from the 3c1 silent copy, and 1c1 was the donor for regions 15 and 16, the output would only include 3c1 because it was the most common silent copy used across the whole sequence. Therefore, this second scenario would be misclassified as only one recombinant based on the current algorithm. However, these exceptions are rare and do not change the overall conclusions drawn from the sequence analysis.

As found with 2c1/6c1 variants discussed above, other recombination events also involved the HV_T_. Many HV_T_ variant sequences were mosaic sequences, containing sequences that mostly matched the parental 1-81-S2 sequence, but also had a few nucleotides that would have been donated from 2c1/6c1. These mosaics were common in strains undergoing pilin Av but were never seen in the Av-deficient control samples. We propose that these mosaic sequences are the result of one strand of a silent copy annealing with the 1-81-S2 *pilE* tail during the process of pilin Av. Alternatively, they could be formed as regions of heteroduplex when a Holliday junction is formed. If one strand of DNA is from 1-81-S2 and the other from a silent copy, there are regions upstream and downstream of the HV_T_ with homology and throughout the HV_T_, the duplex will contain mismatches. The mismatch correction system could then correct the mismatches to either parent or silent variant sequence creating a mosaic tail. We have previously shown that the RuvABC enzyme that processes Holliday junctions is necessary for pilin Av (56) and that mismatch correction controls the frequency of pilin Av (57).

Currently, SwitchAmp does not precisely identify recombination events where two different silent copies donate sequence at different locations in the gene, although the program output can be manually examined to find amplicon sequences representing double recombinants. Additionally, the assignment of silent copy donors can be ambiguous as there are many regions of microhomology among silent copies, if there is a recombination event in one of these regions, the program can detect the variation, but cannot assign a specific donor copy. In this instance, the program provides a list of all possible silent copies that match the varying sequence at each position. These regions are tallied as multidonor in the table.

### Nonpiliated (P^−^) colony morphology affects pilin Av frequencies.

Pilin Av events can result in a colony morphology change when a premature stop codon is incorporated into the coding sequence. Additionally, some combination of silent sequences can produce a nonproductive PilE protein that cannot efficiently assemble into the pilus fiber or create a stable pilus fiber leading to a nonpiliated colony phenotype (58). Either of these events can alter the colony morphology and, more importantly, increase the growth rate of N. gonorrhoeae (58, 59). In order to better understand whether pilin Av events leading to a nonpiliated (P^−^) phenotype were overrepresented in some samples and could possibly explain some of the Av frequencies, we first identified P^−^-associated sequences based on previous studies (Table S4) (38, 60). We used SwitchAmp to identify variants containing P^−^-associated sequences and determined the number of potential P^−^ variants as a factor of the total Av variants in each strain (Table 3).

**TABLE 3:** Analysis of potential P^−^ sequences^*a*^

Strain type	Pool A		Pool B		*P* value for A vs B
	No. of Av reads	% P^−^	No. of Av reads	% P^−^	
*recA6* − IPTG	15	0	ND	ND	NA
*recA6* + 1 mM IPTG	186	12	796	32.8	2.2E−05
FA1090	1,285	41.6	909	26.8	9.1E−07
G4 mutant	15	20	ND	ND	NA
FA1090 *garP_−10_*	6	0	ND	ND	NA
FA1090 *garP_−35_*	277	29	254	28	0.93

a We analyzed the number of variants that contained P^−^ sequences and therefore would produce an underpiliated colony and could outgrow during the 22-h growth. The number of P^−^ variants was divided by total variants to obtain the % P^−^. *P* values were calculated by chi-square tests comparing P^−^ read proportions between biological replicates (A versus B). All pairwise comparisons of summed P^−^ reads under each condition were nonsignificant (*P* > 0.05). ND, not determined; NA, not applicable.

10.1128/mSphere.00562-19.4 TABLE S4 Potential P^−^ variant forming sequences. The parental 1-81-s2 *pilE* sequence is displayed in the first row. The Var regions (regions 1 to 37) and nucleotide locations for all the variable regions of *pilE* is below in the next two rows, respectively. Each Var column then contains the sequences from *pilS* copies known to produce a P^−^ colony morphology. Download Table S4, PDF file, 0.1 MB. Copyright © 2019 Ozer et al. 2019 Ozer et al. This content is distributed under the terms of the Creative Commons Attribution 4.0 International license.

Overall, there were differences in the proportion of P^−^ sequences, but the results were inconsistent among replicates so no strong conclusions can be made. One would expect that if there is no growth benefit selection for nonpiliated N. gonorrhoeae, the proportion of P^−^ variants in the total variant population would be similar regardless of Av frequency. In the *recA6* strains in which recombination was induced with IPTG, we saw an increase in pilin Av frequencies between biological replicates (20%). The higher pilin Av frequency correlated with a much higher proportion of P^−^ sequence, which may explain some of the differences in frequencies (Table 3). We would predict that when P^−^ variants arise early during IPTG induction, the proportion of P^−^ variants will be higher and their growth advantage will amplify their representation. This result contrasts with the percentage of P^−^ variants in the FA1090 biological replicates. The two FA1090 replicates have very similar pilin Av frequencies, but there is a 15% difference in percentages of P^−^ variants between the replicates. We would hypothesize that these P^−^ sequence changes occurred late in the 22-h time frame, which does not allow the high-P^−^ population to outgrow before we collected the DNA. Since our analysis pooled many colonies, and we cannot detect a P^−^ variant within a colony, we cannot independently test these ideas. The Sanger sequencing method of pilin Av previously used was able to report the colony morphology for each variant, which is one benefit to that method (38).

### Measuring pilin Av in association with human macrophages.

One of the advantages of this method of measuring pilin Av in a population of bacteria is the ability to do an analysis within a complex biological context. We tested whether we could measure the frequency of pilin Av during infection of macrophages, a cell type encountered during infection (9). Pilin Av has been detected from isolates within the human host (50, 61). Reduced iron availability has been shown to increase Av frequencies (62), but no other external signal has been found to influence pilin Av rates. Pilin Av frequencies do not change when N. gonorrhoeae infects T84 epithelial cells (63); however, pilin Av has never been measured in the context of macrophage infection.

**FIG 4:**
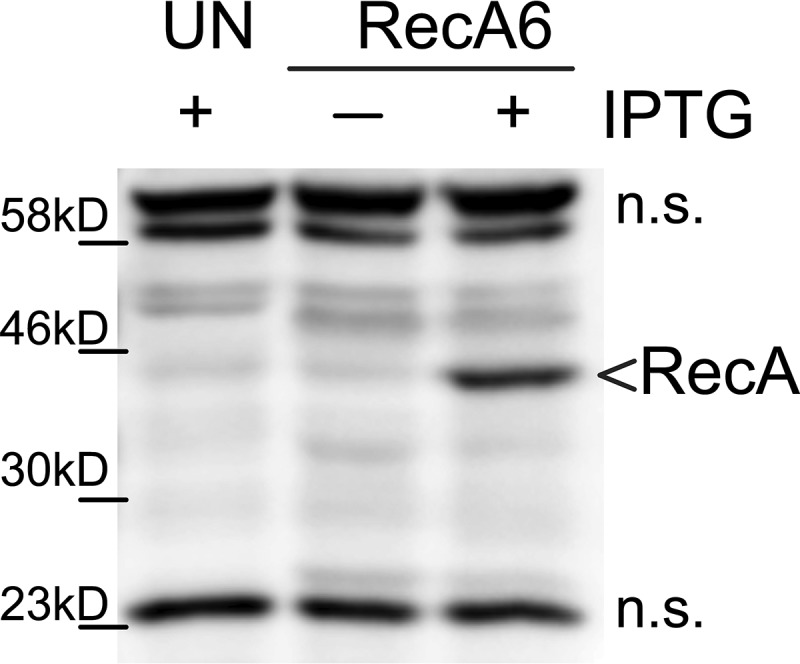
Analysis RecA induction during macrophage infection. Western blot using a polyclonal RecA antibody. Upon addition of IPTG, the RecA band at 40 kDa appears. Based on previous results, the faint band at 40 kDa in the –IPTG lane is another protein. The cross-reactive bands are not significantly (n.s.) different between induced and uninduced strains. Ladder labeled for reference.

**FIG 5:**
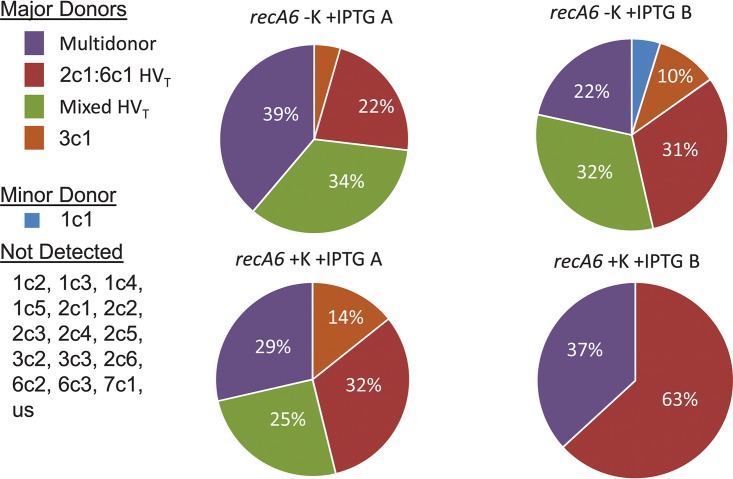
Silent copy choice during macrophage pilin Av. Each pie graph represents all variation events for each of the samples that contained pilin variants during macrophage infection. There are four major donor silent copies in most strains, the HV_T_ change to 2c1 or 6c1, where the exact donor cannot be determined because both donors are identical in that region. The creation of a mosaic tail sequence also originated from 2c1 or 6c1. “Multidonor” encompasses variants that have changes that match multiple potential donors, or there was no single major donor such as a double recombination event. There were only 19 to 125 variation events recorded in each sample. A and B refer to the biological duplicates of pools A and B for each condition.

The FA1090 1-81-S2 *recA6* strain was added to differentiated U937 macrophages at a multiplicity of infection (MOI) of 0.2 with IPTG in the tissue culture medium, and total DNA was isolated after 12 h of infection. IPTG induction of *recA* was confirmed by immunoblot analysis using anti-RecA antiserum (Fig. 4). Potassium was also added to the medium to dampen the macrophage inflammasome response. The *pilE* gene was amplified from the total DNA after 12 h of association. Because the inoculum was prepared in the absence of IPTG, as expected Av was not observed when RecA expression was not induced. Conversely, IPTG-treated N. gonorrhoeae-macrophage cocultures produced pilin Av frequencies of 1.2% and 1.68% in two biological replicates (Table 4). When potassium was added in the medium to dampen the macrophage inflammasome response, the Av frequencies were slightly reduced to 1.1 and 0.69%, respectively, which is significantly lower than without potassium as determined by chi-square analysis (Table 4). The spectra of silent copy donors in macrophage infections and *in vitro* cultures were similar (see Fig. 5 compared to Fig. 3). However, due to the small number of variation events (between 19 and 125), the proportion of each event is different than those measured in monoculture (Fig. 5). Based on this initial analysis, N. gonorrhoeae do vary in the presence of macrophages and silent copy choice is similar to plate-grown bacteria indicating that the silent copies used during plate mirror those that occur during infection.

**TABLE 4:** Pilin Av frequencies during macrophage infection^*a*^

Strain type	Pool A		Pool B		*P* value for A vs B	Total		*P* value vs:	
	No. of reads	% Av	No. of reads	% Av		No. of reads	% Av	FA1090	*recA6* + 1 mM IPTG
Inoculum	6,405	0	7,972	0.1	0.029	14,377	0.06	NA	NA
*recA6* – IPTG	7,350	0	5,367	0	NA	12,717	0	0.11	NA
*recA6* + IPTG	5,666	1.2	7,312	1.68	0.018	12,978	1.46	1.4E−41	NA
*recA6* + K − IPTG	5,049	0	7,614	0	NA	12,663	0	0.11	NA
*recA6* + K + IPTG	8,294	1.1	2,741	0.69	0.086	11,035	0.99	2.0E−26	0.006

a The Av frequencies (% Av) were calculated by dividing the number of variant reads by the total reads after filtering by the SwitchAmp program. All variant strains were analyzed in biological replicates (repeats A and B). *P* values were calculated by chi-square tests comparing Av read proportions between biological replicates (A versus B) or proportions of Av reads between summed totals of reads relative to the inoculum condition (versus inoculum) or relative to the 2 mM K^−^ condition (versus *recA6* + 1 mM IPTG).

This is the first detection of pilin Av during macrophage infection and proves this method can work as a measure of pilin Av when colony morphology cannot be observed. Both biological replicates are similar at this 12-h time point. In the future, we can use this method to determine if the frequency changes during infection, but this would require the number of generations to match from plate-grown to infected bacteria and account for some bacterial death during infection. The addition of potassium did slightly lower the Av frequencies; however, more experiments are needed to determine whether there is a true effect of potassium addition on Av frequencies and whether inflammasome activation could influence the frequency. Regardless, this analysis is the first to show Av does occur in macrophage-associated N. gonorrhoeae and paves the way for future experiments investigating pilin Av in the context of infection.

### Conclusions.

PacBio, long-read amplicon sequencing is an effective means to analyze N. gonorrhoeae pilin Av frequencies. This method will be most useful for strains that grow at different rates or when growth rates cannot be accurately calculated, such as during infection. This method is also conducive to testing many conditions at one time because of the large number of reads produced by the PacBio Sequel instrument. This methodology may not be conducive to testing one strain or condition due to time and cost constraints. In contrast to short-read sequencing approaches, this method reports the spectrum of silent copy donor in addition to the total number of variation events. To make this method adaptable to other scenarios, we created the SwitchAmp program to report pilin Av frequencies. As discussed in the introduction, there are many systems that undergo gene diversification, such as *Borrelia*, trypanosomes, or even antibody generation. PacBio sequencing with the SwitchAmp program allows for the user to input all variable regions and possible sequence changes and the program then analyzes all amplicon reads, so it could be used to analyze many different gene diversification systems.

## MATERIALS AND METHODS

### Strains and growth conditions.

Bacterial strains used in this study were derivatives of the FA1090 clinical isolate. All gonococcal strains were screened for the human challenge isolate 1-81-S2 expressing a particular variant of the type IV pilus (50). N. gonorrhoeae strains were maintained on gonococcal medium base (Difco) modified with Kellogg supplements as described previously (64). FA1090 G4 mutant, *gar_−10_* and *gar_−13_* were constructed as described in reference 33.

### Macrophage infections.

The human monocyte cell line U937 (ATCC CRL-1593.2) was cultured in RPMI 1640 medium (VWR) supplemented with 10% FBS at 37C and 5% CO_2_. For macrophage differentiation, 1 × 10^6^ U937 monocytes were seeded per single well of a 6-well plate and were cultured in the RPMI (plus 10% FBS) supplemented with 10 ng/ml phorbol 12-myristate 13-acetate (PMA) for 24 h. After that period, the medium was replaced with PMA-free medium, and the cells were cultured subsequently for an additional 48 h.

Differentiated macrophages were infected with the N. gonorrhoeae FA1090 *recA6* strain that carries *recA* under an isopropyl-β-d-1-thiogalactopyranoside (IPTG)-inducible promoter (65). The inoculum was prepared by selecting and streaking 4 to 5 piliated colonies as heavy patches on GCB solid medium (Criterion) for 20 h at 37°C and 5% CO_2_. Patches were collected in K^+^-free PBSG (PBS supplemented with 7.5 mM glucose, 0.9 mM CaCl_2_, and 0.7 mM MgCl_2_), and the number of bacteria was determined by optical density measurements.

All infections were completed in PBSG either containing or lacking potassium. Under some experimental conditions, RecA expression was induced by adding 2 mM IPTG at the start of the infection, and expression was validated by lysing the macrophages with Laemmli buffer (2% SDS, 10% glycerol, 0.002% bromophenol blue, 100 mM dithiothreitol [DTT], 125 mM Tris-Cl [pH 6.8]) and probing the total lysates by immunoblot analysis with a polyclonal RecA antibody (generous gift from Michael Cox, University of Wisconsin—Madison) (52) for RecA expression. The inoculum was set at an MOI of 0.2, and viable CFU were confirmed by plating serial dilutions at the beginning (*t* = 0 h) and the end (*t* = 12 h) of the infection.

After the inoculum was added to the macrophage cultures, plates were centrifuged at 1,000 rpm for 5 min to bring the bacteria in contact with the macrophages. At 12 h postinfection, ∼4 × 10^8^ bacterial CFU were recovered by directly lysing the eukaryotic cells with 0.05% Tween 20 (5 min, 37°C) and pelleting the bacteria by centrifugation (13,000 rpm for 5 min). Bacterial pellets were frozen and stored at –20°C. Equivalent numbers of bacteria were also prepared from the inoculum (∼5 × 10^8^ CFU) and frozen. For *pilE* Av frequency analysis, genomic DNA was isolated from the frozen pellets with GenElute bacterial genomic DNA kit (Sigma-Aldrich).

### Preparing strains for PCR amplification and sequencing.

Strains were struck out from frozen stocks and grown overnight for 18 h. All variable strains were grown in duplicate from the freezer. A single colony was picked using a sterile 6-mm filter disk and dispersed in 500 μl GCBL by vortexing. The isolated colony was diluted in GCB medium, and different dilutions were plated on GCB solid medium to obtain 200 to 400 colonies per plate. The remainder of the cell suspension was pelleted and then washed with 1× PBS, and bacteria were lysed in cell lysis buffer. The lysed bacteria were then used as a template for PCR and subsequent sequencing with primers PilRBS and Sp3A. This step ensured that the strains all started as the same *pilE* sequence (the *pilE* allele 1-81-S2) (50).

The colonies were grown for 22 h, and number of colonies was recorded. At least 1,000 colonies (from 1,041 to 1,255 colonies) were pooled in GCBL, and genomic DNA was isolated using Qiagen QiaAmp kits. The genomic DNA was used as a template for PCR and subsequent sequencing with primers PilRBS and Sp3A to determine whether the majority starting sequence was retained (50). Genomic DNA was amplified using the following reaction: 1 ng genomic DNA, 20 μM deoxynucleoside triphosphates (dNTPs), 1× Phusion reaction buffer, 0.5 μM primer 1, 0.5 μM primer 2, 3% DMSO, 1 U Phusion Hot Start Flex (NEB) polymerase, and double-distilled water (ddH_2_O) (Table S1). The reaction was run under the following conditions: 98°C for 30 s for initial denaturation and polymerase activation, 98°C for 10 s, and 65°C for 30 s, then 0.3°C reduced in each cycle, 72°C for 1 min, repeat cycles for 30 times, and final extension for 5 min. For each sample, a different 16-base barcode was used on both the forward and reverse primers, PilRBS (TTTCCCCTTTCAATTAGGAG) and OpaERev (GGGTTCCGGGCGGTGTTTC) leading to a 788-bp product, or 820 bp with barcodes (Table S1).

The PCR products were run on an agarose gel without ethidium bromide and UV exposure. Gel extraction was performed with the QiaQuick Qiagen gel extraction kit, but with the gel slices dissolved at room temperature to maintain DNA integrity. The columns were eluted with Tris-EDTA (TE) buffer and pooled to obtain 300 ng of DNA per sample (Fig. 2). Samples were then submitted to University of Maryland Genomics Resource Center, where the amplicons were purified with SPRI clean up, quantified, and combined into two pools. SMRTbell library prep was performed, and the pools were sequenced on Pacific Biology Sequel SMRT cells with v3 reagents.

### Processing reads and aligning pilin variants.

PacBio subreads generated for each amplicon were converted to Circular Consensus (CCS) reads using ccs v3.0.0 and demultiplexed using the SMRT Analysis software module (Pacific Biosciences, San Francisco, CA). CCS reads for each amplicon pool were then filtered and analyzed using the software package SwitchAmp reported here. The SwitchAmp program is written in Perl and C. The program is run from the command line and compatible with Macintosh and Linux operating systems. Inputs are the fastq-formatted PacBio CCS read file of amplicon sequences for an experiment and a file listing the positions of variable regions relative to the parental sequence and the sequences of the silent copies at these positions (Table S3). This variable region file can be generated manually or from an alignment of the parental sequence to the silent sequences using the script fasta_alignment_conserved.pl that is provided with SwitchAmp. Briefly, SwitchAmp performs the following steps. (i) It filters and removes flanking sequences in each read surrounding the *pilE* gene sequence. (ii) It orients amplicon sequences and groups reads with identical sequences. (iii) It aligns read sequences to the parental sequence using the Needleman-Wunsch algorithm. (iv) It parses variable regions in aligned read sequences to identify matches against the provided parental or silent sequences for each variable region. (v) If a variable region does not perfectly match a provided parental or silent sequence, parental or silent copy sequences with the smallest Levenshtein distance to the read sequence will be determined. (vi) If the total distance from the parental sequence across all variable regions in a read is less than or equal to a given cutoff, all reads with this sequence will counted as parental. (vii) Pairwise Levenshtein distances are calculated for concatenated sequences of all variable regions in each unique read sequence, and hierarchical clustering is performed (Fig. 2). The outputs of the program include a table with each unique read sequence, its total frequency in the input read file, and the closest-matching silent copies in each variable region. The table also shows the Levenshtein distances between each read sequence and the closest-matching silent copy or copies in each variable region. Other program outputs include a file with all unique read sequences, a fasta-formatted file for each variable region listing all variable sequences identified, a newick-formatted tree file with the dendrogram generated by hierarchical clustering of all variable region sequences, and a file summarizing the results of each filtering and processing step by the program. All statistical calculations were performed in R version 3.5.2. Fractions of Av reads or P^−^ reads in each biological condition were compared using the Pearson’s chi-square test. To compare Av or P^−^ read proportions between conditions, pairwise Pearson’s chi-square tests were performed and *P* values were adjusted using Bonferroni correction.

### Data availability.

SwitchAmp and associated software can be found at https://github.com/egonozer/switchAmp with documentation. The raw sequencing reads are available upon request. The amplicon sequencing data are available through the NCBI Sequencing Read Archive (SRA [https://www.ncbi.nlm.nih.gov/sra]) under SRA study accession no. SRP214219. Accession numbers for individual read sets are given in Table S2.
