# Supplementary material for: PacBio Amplicon Sequencing Method To Measure Pilin Antigenic Variation Frequencies of Neisseria gonorrhoeae
Source: mSphere. 2019 Oct 2;4(5):e00562-19. doi: 10.1128/mSphere.00562-19 (PMC6796969; doi:10.1128/mSphere.00562-19)
Supplement: TABLE S1 [file mSphere.00562-19-st001.pdf]

Supplemental Table S1. Primers used in this study.

Pool name	strain and condition	PilRBS	TTTCCCCTTTCAATTAGGAG	SP3A	CCGGAACGGACGACCCCG
20a	*recA6* 0 IPTG 22 hrs	bc1031pilRBS	GATGTCTGAGTGTGTGTTTCCCCTTTCAATTAGGAG	bc1031OpaERev	GATGTCTGAGTGTGTGGGGTTCCGGGCGGTGTTTC
23a	*recA6* 1 mM IPTG 22 hrs	bc1034pilRBS	ATGTGTATATAGATATTTTCCCCTTTCAATTAGGAG	bc1034OpaERev	ATGTGTATATAGATATGGGTTCCGGGCGGTGTTTC
20b	*recA6* 1 mM IPTG 22 hrs	bc1036pilRBS	GAGACACGTCGCACACTTTCCCCTTTCAATTAGGAG	bc1036OpaERev	GAGACACGTCGCACACGGGTTCCGGGCGGTGTTTC
1a	FA1090 grown for 22 hrs	bc1002pilRBS	ACACACAGACTGTGAGTTTCCCCTTTCAATTAGGAG	bc1002OpaERev	ACACACAGACTGTGAGGGGTTCCGGGCGGTGTTTC
1b	FA1090 grown for 22 hrs	bc1009pilRBS	ACACACGCGAGACAGATTTCCCCTTTCAATTAGGAG	bc1009OpaERev	ACACACGCGAGACAGAGGGTTCCGGGCGGTGTTTC
23b	FA1090 G4 mutant 22 hrs	bc1040pilRBS	TGTCATATGAGAGTGTTTTCCCCTTTCAATTAGGAG	bc1040OpaERev	TGTCATATGAGAGTGTGGGTTCCGGGCGGTGTTTC
31b	FA1090 *garP* $_{-10}$ 22 hrs	bc1006pilRBS	CATATATATCAGCTGTTTTCCCCTTTCAATTAGGAG	bc1006OpaERev	CATATATATCAGCTGTGGGTTCCGGGCGGTGTTTC
33a	FA1090 *garP* $_{-35}$ 22 hrs	bc1049pilRBS	ACACGTGTGCTCTCTCTTTCCCCTTTCAATTAGGAG	bc1049OpaERev	ACACGTGTGCTCTCTCGGGTTCCGGGCGGTGTTTC
33b	FA1090 *garP* $_{-35}$ 22 hrs	bc1020pilRBS	CACGACACGACGATGTTTTCCCCTTTCAATTAGGAG	bc1020OpaERev	CACGACACGACGATGTGGGTTCCGGGCGGTGTTTC
3a	Macrophage *recA6* inoculum	bc1006pilRBS	CATATATATCAGCTGTTTTCCCCTTTCAATTAGGAG	bc1006OpaERev	CATATATATCAGCTGTGGGTTCCGGGCGGTGTTTC
19a	Macrophage *recA6* - K 0 IPTG 12 hrs	bc1029pilRBS	TATATATGTCTATAGATTTCCCCTTTCAATTAGGAG	bc1029OpaERev	TATATATGTCTATAGAGGGTTCCGGGCGGTGTTTC
30a	Macrophage *recA6* -K +IPTG 12 hrs	bc1046pilRBS	GATATATCGAGTATATTTTCCCCTTTCAATTAGGAG	bc1046OpaERev	GATATATCGAGTATATGGGTTCCGGGCGGTGTTTC
31a	Macrophage *recA6* +K 0 IPTG 12 hrs	bc1047pilRBS	TGTCATGTGTACACACTTTCCCCTTTCAATTAGGAG	bc1047OpaERev	TGTCATGTGTACACACGGGTTCCGGGCGGTGTTTC
32a	Macrophage *recA6* +K + IPTG 12 hrs	bc1048pilRBS	GTGTGCACTCACACTCTTTCCCCTTTCAATTAGGAG	bc1048OpaERev	GTGTGCACTCACACTCGGGTTCCGGGCGGTGTTTC
27b	Macrophage *recA6* inoculum	bc1048pilRBS	GTGTGCACTCACACTCTTTCCCCTTTCAATTAGGAG	bc1048OpaERev	GTGTGCACTCACACTCGGGTTCCGGGCGGTGTTTC
28b	Macrophage *recA6* - K 0 IPTG 12 hrs	bc1049pilRBS	ACACGTGTGCTCTCTCTTTCCCCTTTCAATTAGGAG	bc1049OpaERev	ACACGTGTGCTCTCTCGGGTTCCGGGCGGTGTTTC
29b	Macrophage *recA6* -K +IPTG 12 hrs	bc1002pilRBS	ACACACAGACTGTGAGTTTCCCCTTTCAATTAGGAG	bc1002OpaERev	ACACACAGACTGTGAGGGGTTCCGGGCGGTGTTTC
30b	Macrophage *recA6* +K 0 IPTG 12 hrs	bc1003pilRBS	ACACATCTCGTGAGAGTTTCCCCTTTCAATTAGGAG	bc1003OpaERev	ACACATCTCGTGAGAGGGGTTCCGGGCGGTGTTTC
32b	Macrophage *recA6* +K + IPTG 12 hrs	bc1007pilRBS	TCTGTATCTCTATGTGTTTCCCCTTTCAATTAGGAG	bc1007OpaERev	TCTGTATCTCTATGTGGGGTTCCGGGCGGTGTTTC

Footnote: Primers with PacBio barcodes for each strain and condition are listed.
