# Supplementary material for: PacBio Amplicon Sequencing Method To Measure Pilin Antigenic Variation Frequencies of Neisseria gonorrhoeae
Source: mSphere. 2019 Oct 2;4(5):e00562-19. doi: 10.1128/mSphere.00562-19 (PMC6796969; doi:10.1128/mSphere.00562-19)
Supplement: TABLE S2 [file mSphere.00562-19-st002.pdf]

Supplemental Table S2: Read Statistics

											Up-/downstream sequence filtering		Group amplicons	Remove reads with ≥ 2 differences from		Remove amplicons with fewer than 3	
**Sample name**	**Experiment**	**Replicate #**	**Library ID**	**SRA study**	**SRA accession**	**BioProject accession**	**BioSample accession**	**# Reads**	**# Bases**	**Mean read length**	**# Reads**	**# Unique**	**# Unique**	**# Reads**	**# Unique**	**# Reads**	**# Unique**
35mutFA_22h_a	FA1090 *garP* $_{-35}$ 22 hrs	1	33a	SRP214219	SRR9670522	PRJNA553228	SAMN12255025	5972	4717392	789.92	5283	1110	644	4837	328	4522	42
35mutFA_22h_b	FA1090 *garP* $_{-35}$ 22 hrs	2	33b	SRP214219	SRR9670511	PRJNA553228	SAMN12255041	6011	4577275	761.48	5151	1095	605	4708	291	4433	29
FA-10mut_22h	FA1090 *garP* $_{-10}$ 22 hrs		31b	SRP214219	SRR9670517	PRJNA553228	SAMN12255039	6015	4759857	791.33	4788	480	79	4768	59	4707	3
FA1090_22h_a	FA1090 grown for 22 hrs	1	1a	SRP214219	SRR9670503	PRJNA553228	SAMN12255012	10842	8628492	795.84	9752	2824	2004	8130	886	7296	109
FA1090_22h_b	FA1090 grown for 22 hrs	2	1b	SRP214219	SRR9670527	PRJNA553228	SAMN12255026	8690	6941742	798.82	7840	2492	1945	6220	825	5396	71
G4mutFA1090_22h	FA1090 G4 mutant 22 hrs		23b	SRP214219	SRR9670514	PRJNA553228	SAMN12255034	8757	6925157	790.81	7433	744	170	7354	102	7252	5
RecA6-IPTG_22h	*recA6* 0 IPTG 22 hrs		20a	SRP214219	SRR9670509	PRJNA553228	SAMN12255020	10227	8158624	797.75	9092	935	205	9034	153	8881	4
RecA6+IPTG_22h_a	*recA6* 1 mM IPTG 22 hrs	1	23a	SRP214219	SRR9670510	PRJNA553228	SAMN12255021	5141	4090877	795.74	4555	920	472	4232	232	4001	18
RecA6+IPTG_22h_b	*recA6* 1 mM IPTG 22 hrs	2	20b	SRP214219	SRR9670515	PRJNA553228	SAMN12255033	15315	11909036	777.61	13220	2135	1105	12416	562	11884	65
-K-IPTG_Mac_a	Macrophage *recA6* - K 0 IPTG 12 hrs	1	19a	SRP214219	SRR9670506	PRJNA553228	SAMN12255019	8412	6700755	796.57	7437	711	86	7422	71	7350	1
-K-IPTG_Mac_b	Macrophage *recA6* - K 0 IPTG 12 hrs	2	28b	SRP214219	SRR9670520	PRJNA553228	SAMN12255036	6166	4842998	785.44	5445	548	77	5433	65	5367	1
-K+IPTG_Mac_a	Macrophage *recA6* -K +IPTG 12 hrs	1	30a	SRP214219	SRR9670523	PRJNA553228	SAMN12255022	7108	5609835	789.23	6006	845	260	5854	132	5733	15
-K+IPTG_Mac_b	Macrophage *recA6* -K +IPTG 12 hrs	2	29b	SRP214219	SRR9670519	PRJNA553228	SAMN12255037	8983	6984210	777.49	7887	1047	407	7649	214	7437	20
+K-IPTG1_Mac_a	Macrophage *recA6* +K 0 IPTG 12 hrs	1	31a	SRP214219	SRR9670524	PRJNA553228	SAMN12255023	5739	4522742	788.07	5116	472	67	5104	55	5049	1
+K-IPTG2_Mac_b	Macrophage *recA6* +K 0 IPTG 12 hrs	2	30b	SRP214219	SRR9670518	PRJNA553228	SAMN12255038	8732	6776041	776	7721	735	107	7701	87	7614	1
+K+IPTG_Mac_a	Macrophage *recA6* +K + IPTG 12 hrs	1	32a	SRP214219	SRR9670521	PRJNA553228	SAMN12255024	10040	7856093	782.48	8786	1127	360	8595	213	8385	13
+K+IPTG_Mac_b	Macrophage *recA6* +K + IPTG 12 hrs	2	32b	SRP214219	SRR9670512	PRJNA553228	SAMN12255040	3542	2763803	780.29	2935	413	162	2854	91	2760	4
Innoc1_Mac_a	Macrophage *recA6* inoculum	1	3a	SRP214219	SRR9670504	PRJNA553228	SAMN12255013	7865	6245248	794.06	6512	631	103	6488	80	6405	1
Innoc1_Mac_b	Macrophage *recA6* inoculum	2	27b	SRP214219	SRR9670513	PRJNA553228	SAMN12255035	9104	7128006	782.95	8095	731	115	8079	100	7980	3

Footnotes: Strain names, accession numbers, reads, and number of sequences filtered during each step of filtering and analysis
