# Supplementary material for: PacBio Amplicon Sequencing Method To Measure Pilin Antigenic Variation Frequencies of Neisseria gonorrhoeae
Source: mSphere. 2019 Oct 2;4(5):e00562-19. doi: 10.1128/mSphere.00562-19 (PMC6796969; doi:10.1128/mSphere.00562-19)
Supplement: TABLE S3 [file mSphere.00562-19-st003.pdf]

Supplemental Table S3: Alignment of pilE and pilS across all variable regions

nt position	160	162	163	167-168	173	176	180-181	187-188	195-199	205-210	223-234	239	253	255-258	262-264	267-273	282	289-290	295-298	301-302	304-305	315	322	326	332	347	350	355	358-359	364	394-395	400-441	445	447	452	474	477-492
1c1	G	T	G	GG	G	C	TC	AC	GAAAG	GGTGAT	TCCCCCGCCGACAAA	A	C	GAAA	GAA	CGCAAAA	T	GA	AAAC	AG	GG	A	A	G	A	C	A	G	GA	T	AA	GACGCCGGCGCCAAAGCCGACGACGTCAAAGCCGACGCCGCCAACGCC	G	A	A	C	TGAATCATCTGCCACC
1c2	G	T	A	GG	G	C	TC	AA	GAAAG	ACTTCT	TCCCCCGCCGAA	A	A	AAGC	ACG	CGCAAAA	T	CA	AATC	AG	GG	T	A	A	A	C	A	G	AA	T	AC	AACGACGCCAAAGCCGACGCCAAAGACGACACCGTCACCGCC	G	A	A	C	TGAATCATCTGCC
1c3	G	T	G	GG	G	C	TC	AA	GGAAA	ACTTCT	TCCCCCCCCTCCGAC	A	A	AGAG	GAA	TAAAAAC	T	AC	CTTT	AG	GG	T	A	G	A	C	G	G	AA	T	AC	GCCAAAGCCGACGCCGACGCCGACGCCGCCGGCAAAGACACCACCAAC	G	C	A	C	TGAATCATCTGCCGTT
1c4	G	T	G	GG	G	T	TC	GA	GGAAG	ACTTCT	TCCCCCCCCACCGAC	A	A	AAGC	ACG	CGCAAAA	C	GA	GCTT	AC	GG	T	A	G	A	C	A	C	GA	-	AA	GACGCCGGCGCCAAAACCGGCGCCGACGACGTCAAAGCCGACGGCAAAGACACCGACAAA	A	C	A	C	TAAATCATCTGCCGTT
1c5	G	T	A	AG	A	T	TC	GA	CAAAG	GGCTCT	TCCGCTTCAAAA	T	A	GGAA	AAA	CGAAAAC	C	CA	GCTT	AG	AA	A	A	A	A	C	A	G	AA	T	AA	ACCGAAGCCAACGCCAAAGCCGGCACCGACGACGTCGCCAAAGACGACACCGCCGGCACCAAA	G	C	G	C	TGAATCATCG
2c1	G	C	A	AA	A	C	TA	GA	CGCCG	GGCGCT	TCCGCTTCAACA	A	C	GAAA	GAA	CGCAAAA	T	CA	GCTT	AC	GG	A	C	A	A	C	A	C	GA	T	AC	ACCGGCGACAACGACGACACCGTTGCCGACGCCAACAACGCC	G	C	A	C	TAAACATGATGCCAAA
2c2	G	T	A	AG	G	C	TC	GA	GAAAG	GACAAG	TCCCCCCCCTCCAAC	A	G	AAGC	ACG	CACAAAC	T	AC	CTTT	AG	GG	T	A	G	A	C	A	C	GC	T	CA	GCCAAAGCCGACGACGCCGTCACCGCCGACGCCAACAACGCC	G	C	A	T	TACGTCATCTGCCAAA
2c3	G	T	A	AG	A	T	TC	AT	GGAAA	GACAAG	TCTTCTTCATCA	A	A	GGAA	AAA	CGAAAAC	C	AC	AATT	AG	AA	A	A	A	G	C	A	G	AA	T	AA	GCCAACGTTGCCGCCGCCAACGACGACGACGTTACCGACGACAAAAACAACAACGGC	G	C	A	C	TAAATCATCTGCC
2c4	G	T	G	GG	G	T	TC	GA	GGAAG	ACTTCT	TCCCCCCCACCGA	A	A	AAGC	ACG	CGCAAAA	C	GA	GCTT	AC	GG	T	A	G	A	C	A	C	GA	T	AA	GCCGACAACAACGGCAACATTACCGCCGACAACGGCAACGCC	G	A	A	C	TGAATCATCTGCC
2c5	G	T	G	GG	A	T	TC	GA	GGAAA	GACAAG	TCCGCCTCCGAC	A	A	AAGC	ACG	CGCAAAA	T	GA	GCTT	AC	GG	A	C	G	A	C	A	G	AA	T	AA	ACCGAAGCCAACGCCAAAGCCGGCACCGACGACGTCGCCAAAGACGACACCGCCGGCACCAAA	G	C	A	C	TGAATCATCG
2c6	G	T	A	GG	G	C	TC	AT	GAAAG	ACTTCT	TCCTCCGCCGCCGAC	A	C	GAAA	GAA	CAACAAC	T	AC	GCTT	AG	AA	A	C	G	G	C	A	G	AA	-	AC	GACGACAAAGCCAAAGACGACGTCAAAGCCGACGGCACCGCCGGCACCAAA	G	C	A	C	TGAATCATCG
3c1	G	T	G	GG	A	T	TC	GA	GGAAA	ACTTCT	TCCTCCGACAAA	A	C	GAAA	GAA	CGCAAAA	T	CA	GCTT	AC	GG	A	C	G	A	C	A	C	GA	T	AC	AACGACGCCAAAGCCGACGACGTCAAAGCCGACGCCGCCAACGCC	G	A	A	C	TGAATCATCTGCCACC
3c2	C	C	A	AA	A	T	-A	GA	CAAAG	GGCTCT	TCCGCTTCAACA	A	C	GAAA	GAA	CACAAAC	T	CA	GCTT	AC	GG	A	A	G	A	C	G	C	GA	T	AA	GACGACGCCGCCGCCAAAGACGACACCGTCACCGCCGACGCCACCGGCAACGACGGCAAA	G	C	A	C	TAAATCAACTGCCGTT
3c3	G	C	A	AG	A	T	TC	AT	GAAAG	ACTTCT	AACCCCACCGAC	A	G	AAGC	ACG	CACAAAC	T	AA	CTTT	AG	GG	A	C	G	G	C	A	G	GC	T	AA	ACCGAAGCCAACGCCGACGCCGCCGGCAAAGACACCACCAACGGC	A	C	A	C	CCCGTTCTCTGCTAGC
6c1	G	T	A	GG	A	T	TA	AA	CGCCG	GGCGCT	TCCCCCGCCACCGA	A	A	GGAA	AAA	CGAAAAC	C	CA	GCTT	AC	GG	T	A	G	A	C	A	C	GA	T	AA	GACGCCGGCGCCAAAACCGGCGCCGACGACGTCAAAGCCGACGGCAACAACGGC	A	C	A	C	TAAACATGATGCCAAA
6c2	G	T	G	GG	A	C	TC	AA	GGAAG	ACTTCT	TCCCCCCCTCCGAC	A	C	AAGC	ACG	CGCAAAC	T	GA	AAAT	GA	GG	A	A	G	A	G	G	G	AA	T	AA	GACGCCAACAACGCCAACAACGACGCCGTCACCGACGACACCACCGGCAACGGCAACGAAAAA	G	A	A	C	TGAATCATCTGCCGTT
6c3	-	-	-	--	A	C	TA	AA	CGCAA	GGCGCT	TCCGCCTCCGAC	A	G	AAGC	ACG	CGAAAAA	T	AA	CTTT	AG	GG	A	C	G	A	C	A	G	GC	T	AA	AACGACGCCGCCAACGACGACGTTACCGACGACGCCGGCACCGACAACGGCGGCAAAGGCAAA	G	C	A	C	TAAATCAACTGCCGTT
7c1	G	C	A	AG	A	T	TC	AC	GGAAA	ACTTCT	TCCTCCGCCACCGAC	A	C	AAGC	ACG	CGCAAAC	T	GA	AAAT	GA	GG	A	C	G	G	C	G	G	GC	T	AC	GCCAAAGCCAAAGACGCCGACGACGTTACCGACGACGCCGGCACCCACAACGGCGGCAAAGGCAAA	G	C	A	C	TAAATCAACTGCCAAA
us	-	-	-	--	-	-	--	-	-----	GGTGAT	TCCTCCGCCGAA	A	A	AAGC	ACG	CGCAAAC	T	GA	GCTT	AG	GG	A	A	G	G	C	G	C	GA	T	AC	AACGACGCCGCCGACAACGACGACGTCGCCAAAGACGACGCCGCCGGCAACGCC	G	A	A	C	TGAACCAACTGCCACC
1-81-S2	G	C	A	AG	A	T	TC	AT	GAAAG	ACTTCT	TCCGCTTCAACA	A	C	GAAA	GAA	CGCAAAA	T	CA	GCTT	AC	GG	A	C	A	A	C	A	C	GA	T	AC	ACCGGCGACAACGACGACACCGTTGCCGACGCCAACAACGCC	G	C	A	C	TGAATCATCTGCCACC
