# Supplementary material for: PacBio Amplicon Sequencing Method To Measure Pilin Antigenic Variation Frequencies of Neisseria gonorrhoeae
Source: mSphere. 2019 Oct 2;4(5):e00562-19. doi: 10.1128/mSphere.00562-19 (PMC6796969; doi:10.1128/mSphere.00562-19)
Supplement: TABLE S4 [file mSphere.00562-19-st004.pdf]

Supplemental Table S4. Potential P- variant forming sequences.

1-81-S2	G	C	A	AG	A	T	TC	AT	GAAAG	ACTTCT	TCCGCTTCAACA	A	C	GAAA	GAA	CGCAAAA	T	CA	GCTT	AC	GG	A	C	A	A	C	A	C	GA	T	AC	ACCGGCGACAACGACGACACCGTTGCCGACGCCAACAACGCC	G	C	A	C	TGAATCATCTGCCACC
var	1	2	3	4	5	6	7	8	9	10	11	12	13	14	15	16	17			20	21	22	23	24	25	26	27	28	29	30	31	32	33	34	35	36	37
nt location		162				176	180-181	187-188	195-199	205-210	223-234	239	253	255-258	262-264	267-273	282			301-302	304-305	315	322	326				355	358-359			400-441					
		1c5				2c1	2c1	1c4	1c4	1c5	1c3	1c5	1c5	1c5	1c5	1c5	1c5			1c5	1c5	6c1	1c5	6c1				1c5	1c5			1c1					
		6c1					3c2	1c5	1c5	2c1	1c5			6c1	6c1	6c1	6c1						6c1														
							6c1	2c1	2c1	6c1	2c4																										
								2c2	2c4		3c2																										
								2c4	6c1		6c1																										
								2c5	6c2		6c2																										
								3c1			long repeats																										
								3c2			1c3																										
								6c1			1c4																										
											2c2																										

Footnotes: The parental sequence is displayed in the first row. The var region and nt location for all the reigons of *pilE* is below. Each Var column then contains the pilS copies known to cause P- colony morphology at that specific position in the gene.
